# A year of experience with COVID‐19 in patients with cancer: A nationwide study

**DOI:** 10.1002/cnr2.1678

**Published:** 2022-11-27

**Authors:** Mina Khosravifar, Sogol Koolaji, Negar Rezaei, Ali Ghanbari, Seyedeh Melika Hashemi, Erfan Ghasemi, Ali Bitaraf, Ozra Tabatabaei‐Malazy, Nazila Rezaei, Sahar Mohammadi Fateh, Arezou Dilmaghani‐Marand, Rosa Haghshenas, Ameneh Kazemi, Erfan Pakatchian, Farzad Kompani, Shirin Djalalinia

**Affiliations:** ^1^ Non‐Communicable Diseases Research Center, Endocrinology and Metabolism Population Sciences Institute Tehran University of Medical Sciences Tehran Iran; ^2^ Endocrinology and Metabolism Research Center, Endocrinology and Metabolism Clinical Sciences Institute Tehran University of Medical Sciences Tehran Iran; ^3^ School of Medicine Kermanshah University of Medical sciences Kermanshah Iran; ^4^ Division of Hematology and Oncology Children's Medical Center, Pediatrics Center of Excellence, Tehran University of Medical Sciences Tehran Iran; ^5^ Deputy of Research and Technology Ministry of Health and Medical Education Tehran Iran

**Keywords:** cancer, comorbidity, coronavirus disease 2019, malignancy, risk factor

## Abstract

**Background:**

Cancer is a major public health problem and comorbidity associated with COVID‐19 infection. According to previous studies, a higher mortality rate of COVID‐19 in cancer patients has been reported.

**Aims:**

This study was undertaken to determine associated risk factors and epidemiological characteristics of hospitalized COVID‐19 patients with cancer using a nationwide COVID‐19 hospital data registry in Iran for the first time.

**Methods:**

In this retrospective study, we used a national data registry of hospitalized patients with Severe Acute Respiratory Syndrome (SARS) symptoms and patients with confirmed positive COVID‐19 PCR between 18 February 2020 and 18 November 2020. The patients were classified into two groups patients with/without malignancy. Logistic regression model was utilized to analyze demographic factors, clinical features, comorbidities, and their associations with the disease outcomes.

**Results:**

In this study, 11 068 and 645 186 in‐patients with SARS symptoms with and without malignancy were included, respectively. About 1.11% of our RT‐PCR‐positive patients had cancer. In patients with malignancy and COVID‐19, older ages than 60 (OR: 1.88, 95% CI: 1.29–2.74, *p*‐value: .001), male gender (OR: 1.43, 95% CI: 1.16–1.77, *p*‐value: .001), concomitant chronic pulmonary diseases (CPD) (OR: 1.75, 95% CI: 1.14–2.68, *p*‐value: .009), and presence of dyspnea (OR; 2.00, 95% CI: 1.60–2.48, *p*‐value: <.001) were associated with increased mortality rate.

**Conclusion:**

Given the immunocompromised state of patients with malignancy and their vulnerability to Covid‐19 complications, collecting data on the comorbidities and their effects on the disease outcome can build on a better clinical view and help clinicians make decisions to manage these cases better; for example, determining special clinical care, especially in the shortage of health services.

## 1 INTRODUCTION

Since the first report of the coronavirus disease 2019 (COVID‐19) in December 2019, which is caused by severe acute respiratory syndrome coronavirus 2 (SARS‐CoV‐2), the disease has resulted in remarkable mortality and morbidity worldwide.^1^ Regarding variable ranges of disease severity from asymptomatic infections to respiratory failure or death,^2^ several studies have evaluated the importance of underlying comorbidities on the disease severity and outcomes, of which old age, diabetes (DM), cardiovascular diseases (CVDs), hypertension (HTN), and cancer are assumed to be associated with unfavorable prognosis.^3^, ^4^, ^5^ In this line, considering cancer as one of the most common health problems, with more than 18 million incident cases per year worldwide, assessing the risk of severity and mortality in this group has received remarkable attention.^6^, ^7^ Although several studies have reported a higher mortality rate of COVID‐19 in cancer patients,^8^, ^9^ this finding was not observed in all reports.^10^, ^11^ Despite the unknown underlying mechanism of increased mortality in cancer patients, systemic inflammation is indicated as a prognostic domain in infected patients with malignancy for poor outcomes.^12^ Along with the immunocompromised status of patients with cancer, other issues like coronophobia, which may result in delayed diagnosis,^13^ and COVID‐19 burden on the health care prioritization that may compromise treatment in cancer patients,^14^ should be considered as factors affecting the risks of COVID‐19 in these patients.

As far as demographic factors are concerned, the increased number of comorbidities such as HTN, DM, CVDs, older ages, and male gender predispose patients with cancer to a worse mortality rate.^15^, ^16^, ^17^

In addition, it was previously shown that in patients with cancer, the presence of other comorbidities and older ages significantly changes the rate of RT‐PCR positivity, and these subgroups may benefit from routine COVID‐19 testing.^18^ On the other hand, studies have confirmed the high possibility of asymptomatic disease in patients with cancer who were diagnosed with serologic tests lacking a confirmed COVID‐19 history.^19^, ^20^

Given that healthcare resources are limited, understanding the mortality and severity risk factors of the patients who can be at increased risk for COVID‐19 prepares us for potential future attacks of infectious diseases and help us to manage cancer patients better.

Having in mind that cancer is a threatening public health problem, along with controversial reports of these patients' vulnerability to COVID‐19 infection and variability of the disease presentation,^21^ the present study was undertaken to investigate associated comorbidities and epidemiological characteristics of cancer patients experiencing COVID‐19 using a nationwide COVID‐19 data registry in Iran for the first time in hospitalized cancer patients.

## 2 METHODS

### 2.1 Overview

In this retrospective study, a national data registry of hospitalized COVID‐19 patients between 18 February 2020 and 18 November 2020 was used in this study. All hospitalized patients from all age groups with Severe Acute Respiratory Syndrome (SARS) symptoms were diagnosed with COVID‐19 either by positive COVID‐19 RT‐PCR or chest computed tomography (CT) scan manifestations in favor of the disease. The patients were classified into two groups patients with/without malignancy. In addition, a digital portal was designed by the Ministry of Health and Medical Education (MoHME) that was used for data gathering from all private and governmental hospitals in the 31 provinces of Iran. The infection control unit of the hospitals managed the data gathering and transferred them to the portal in a daily process. Questions were completed by the skilled medical staff based on the patients' reported data. For young children from whom taking a medical history was not reliable, parents were asked to complete the questions. In the current study, no inclusion and exclusion data were applied, and all available data were used.

### 2.2 Case definition

The status of malignant disorders was based on a self‐report of active or in‐remission status of neoplasia. According to the physician's decision, patients experiencing conditions like respiratory symptoms, laboratory findings like low or normal white‐cell count with low lymphocyte count, radiographic findings in favor of SARS‐CoV‐2 pneumonia, history of social contact with suspicious people for COVID‐19 in the past 14 days, were hospitalized with the diagnosis of SARS. If these patients' nasopharyngeal and oropharyngeal COVID‐19 Real‐Time Reverse Transcription Polymerase Chain Reaction (RT‐PCR) test were positive, they were considered as confirmed cases.

### 2.3 Data collection

The data were collected and completed by medical staff using electronic forms from Hospital Information System (HIS). Demographic characteristics consist of age and gender (male/female), self‐reported medical history of comorbidities, including CVDs (including ischemic heart disease and heart failure), DM (including Type1, type2, and gestational diabetes), kidney disease (including chronic kidney disease, end‐stage renal disease), liver disease (including cirrhosis), chronic pulmonary diseases (CPD) (including, chronic obstructive pulmonary disease [COPD], asthma, and interstitial lung diseases), and immune deficiency disease (IDD) (including, congenital and acquired immunodeficiency diseases except for cancer and chemotherapy). In addition, signs and symptoms including cough, dyspnea, myalgia, diarrhea, sore throat, and headache; COVID‐19 PCR diagnostic test result; the status of pregnancy; intensive care unit (ICU) admission, ventilation assistance (including invasive and non‐invasive mechanical ventilation); and clinical outcome, including recovery, in treatment, and death were recorded.

### 2.4 Statistical analysis

#### 2.4.1 Data cleaning and descriptive statistics

**FIGURE 1:**
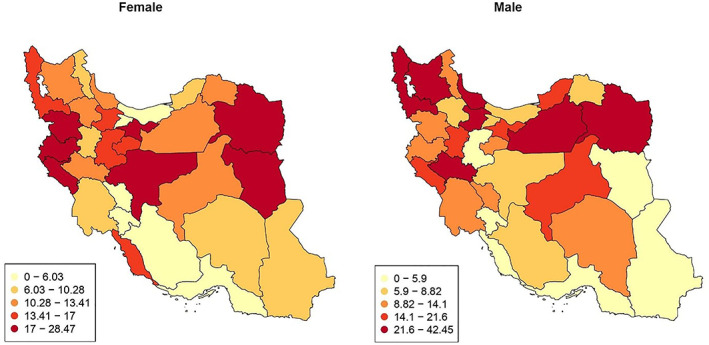
The geographical distribution of Covid‐19 mortality in patients with malignancy among provinces showing a diverse pattern in the country. Specifically, a higher mortality rate was seen in the North‐East border provinces

After the data cleaning process, including managing duplicate cases and recoding variables, the data on demographic factors, comorbidities, signs and symptoms, and patients' outcomes were described in frequency tables. Continuous variables (age and body temperature) did not have a normal distribution, so we reported a median ± interquartile range of 5%–95%. The geographical distribution of COVID‐19 mortality rates among patients with malignancy was illustrated on a map (Figure 1).

#### 2.4.2 Analytical statistics

Independent samples *T*‐test and logistic regression were used for statistical analysis. The frequency of different factors between malignant and non‐malignant patients was compared with independent samples *T*‐test. To assess the correlation between demographic characteristics, comorbidities, and symptoms as independent variables and mortality as the outcome variable, univariate logistic regression was used, and the crude odds ratio (OR) was calculated with a 95% confidence interval (95% CI). We used a backward stepwise selection method to build multiple logistic regression models, including the independent variable groups of demographic factors, comorbidities, and clinical features. Adjusted ORs (adj. ORs) with *p*‐values of less than .05 and 95% CIs were reported for significant variables associated with outcomes. In the death, multiple logistic models, variables of age and sex were adjusted for comorbidities, ICU admission, and ventilator support. Comorbidities and symptoms were adjusted for age, sex, ventilator support, and ICU admission.

Stata 14 and R version 3.5.2 were used for statistical analysis.

## 3 RESULTS

### 3.1 Demographic features

In this study, 656 254 patients with SARS symptoms who had been admitted to hospitals from February 18 to November 18, 2020, in all 31 provinces of Iran were evaluated. The prevalence of malignancy among our hospitalized SARS and RT‐PCR‐positive patients was 1.68% and 1.11%, respectively. The mean of age in RT‐PCR positive patients with and without malignant disorders was 58.28 years (24–85) and 55.79 years (25–85), respectively. Considering the age groups, among the hospitalized and RT‐PCR positive patients with malignancy included in this report, a greater proportion of them were more than 60 years old compared with patients without malignant disorders (53.03% and 45.86%, respectively, *p*‐value <.001).

Females were slightly more than males in hospitalized and RT‐PCR positive patients with cancer (53.87% and 52.09%, respectively), which was not statistically significant (Table 1).

**TABLE 1:** Characteristics of hospitalized patients with and without malignancy

		Patients with SARS symptoms and malignancy *N* (%) or mean (5%–95%)		Patients with SARS symptoms, without malignancy *N* (%) or mean (5%–95%)		*p*‐value among hospitalized	*p*‐value among RT‐PCR positive
		Total hospitalized (*N* = 11 068)	COVID RT‐PCR positive (*N* = 3016)	Total hospitalized (*N* = 645 186)	COVID RT‐PCR positive (*N* = 268 280)		
Age	<20 years	706 (6.29)	114 (3.75)	41 454 (5.31)	7517 (2.35)	<.001	<.001
	20–40 years	1273 (11.51)	335 (11.11)	125 336 (19.66)	50 352 (18.86)	<.001	<.001
	40–60 years	3337 (30.18)	968 (32.11)	186 021 (29.18)	87 940 (32.93)	<.001	<.001
	>60 years	5752 (52.02)	1599 (53.03)	292 375 (45.86)	122 471 (45.86)	<.001	<.001
Sex (male)		5106 (46.13)	1445 (47.91)	301 444 (46.72)	125 655 (45.84)	.21	.23
Pregnancy		20 (0.54)	5 (1.49)	6132 (3.05)	2075 (2.73)	<.001	<.001
Comorbidities	Diabetes	1215 (10.98)	402 (13.33)	77 346 (11.99)	37 006 (13.79)	.001	.46
	Kidney disease	411 (3.71)	119 (3.95)	17 914 (2.78)	6368 (2.37)	<.001	<.001
	IDD	295 (2.67)	87 (2.88)	2565 (0.40)	975 (0.36)	<.001	<.001
	Liver disease	265 (2.93)	83 (2.75)	4215 (0.65)	1596 (0.59)	<.001	<.001
	CVD	1617 (14.61)	458 (15.19)	106 040 (16.44)	43 847 (16.34)	<.001	.08
	CPD	625 (5.85)	164 (5.62)	26 048 (4.20)	9174 (3.53)	<.001	<.001
Sign and symptoms	Dyspnea	5695 (51.45)	1711 (56.73)	327 489 (50.76)	148 925 (55.51)	.14	.18
	Cough	3931 (35.52)	1284 (42.57)	290 232 (44.98)	136 213 (50.77)	<.001	<.001
	Myalgia	1991 (17.99)	697 (23.11)	146 730 (22.74)	71 544 (26.67)	<.001	<.001
	Headache	806 (7.28)	272 (9.02)	65 573 (10.16)	31 741 (11.83)	<.001	<.001
	Sore throat	696 (6.29)	242 (8.02)	64 939 (10.07)	29 562 (11.02)	<.001	<.001
	Diarrhea	501 (4.53)	154 (5.11)	28 200 (4.37)	11 967 (4.46)	.42	<.001
	Body temp	38 (37–39)	38.10 (37–39)	38.00 (37–39)	38.06 (37–39)	.72	.042
Outcome	ICU admission	1742 (15.74)	440 (14.59)	50 266 (7.79)	18 000 (6.71)	<.001	<.001
	Ventilation support	1366 (19.27)	372 (18.67)	47 592 (12.34)	20 409 (12.17)	<.001	<.001
	Death	2441 (22.05)	873 (28.95)	63 378 (9.82)	35 661 (13.29)	<.001	<.001
	Recovered	5571 (50.33)	1414 (46.88)	376 974 (58.43)	155 046 (57.79)	<.001	<.001
	Under treatment	3056 (27.61)	729 (24.17)	204 800 (31.74)	77 564 (28.91)	<.001	<.001

Abbreviations: CPD, chronic pulmonary disease; CVD, cardiovascular disease; ICU, intensive care unit; IDD, immune deficiency disease.

The geographical distribution of Covid‐19 mortality in patients with malignancy among provinces indicated a diverse pattern in the country. Specifically, a higher mortality rate was seen in the North‐East border provinces (Figure 1).

### 3.2 Comorbidities

Regarding the six comorbidities that were investigated in this study, CVDs had the highest prevalence in hospitalized patients with or without cancer (14.61% vs. 16.44%, *p*‐value <.001). In the following ranks, diabetes comprised 10.98% of hospitalized patients with malignancy and 11.99% of hospitalized patients in the counter group (*p*‐value <.001). However, among patients with COVID‐19 RT‐PCR positive tests, differences in the DM and CVD prevalence between patients with and without malignancy were not significant. The records of the subsequent most prevalent comorbidities from the highest to lowest, including CPD, kidney disease, liver disease, and IDD, were significantly higher in hospitalized and COVID‐19 positive patients with malignancy (all *p*‐values <.001). Additionally, the prevalence of IDD and liver disease in COVID‐19 positive cases were 8 and 4.71 times greater in patients with malignancy compared to the non‐malignant group (Table 1).

### 3.3 Signs and symptoms

Among six signs and symptoms that were enrolled in the study, dyspnea (around 40%) was the most frequent one in both study groups. However, regarding dyspnea, no significant differences were observed between either of the study groups. The results showed that the prevalence of cough, myalgia, headache, and sore throat was significantly higher in patients without malignancy in both groups of hospitalized (*p*‐value <.001) and COVID‐19 positive cases (*p*‐value <.001) (Table 1).

### 3.4 Outcome

Of hospitalized patients and COVID‐19 RT‐PCR positive patients with malignancy, 22.05% and 28.95% died, respectively, which was significantly greater than patients without malignancy (*p*‐values <.001). In addition, the rates of ventilation support (*p*‐value <.001) and ICU admission (*p*‐value <.001) were significantly higher in patients with malignancy (Table 1).

### 3.5 Logistic regression analysis of associated factors with mortality among patients with malignancy and positive RT‐PCR

In cases with malignancy and positive COVID‐19 RT‐PCR test, the adjusted odds of mortality in patients aged more than 60 was 1.88 (95% CI: 1.29–2.74, *p*‐value: .001) times higher than the 20–40‐year‐old group. Moreover, males' adjusted odds of mortality was 1.43 (95% CI: 1.16–1.77, *p*‐value: .001) times more than females. Regarding the included comorbidities, only CPD was associated with a 1.75 (95% CI: 1.14–2.68, *p*‐value: .009) times greater mortality rate in multivariate analysis for COVID‐19 patients with malignancy. Among included symptoms, adjusted mortality rates in these patients were higher in patients with dyspnea (OR; 2.00, 95% CI: 1.60–2.48, *p*‐value: <.001) and lower in patients with cough (OR; 0.75, 95% CI: 0.61–0.93, *p*‐value: .011) (Table 2).

**TABLE 2:** Odds ratio of mortality in patients with malignancy and positive COVID‐19 RT‐PCR

Demographic		Patients with malignancy with positive COVID‐19 RT‐PCR (*n* = 3016)			
		OR crude (CI 95%)	PV	OR adjusted (CI 95%)	PV
Age	20–40	Reference	‐	Reference	‐
	<20	0.85 (0.49–1.47)	0.56	0.91 (0.45–1.86)	0.81^a^
	40–60	1.31 (0.97–1.78)	0.07	1.20 (0.81–1.78)	0.36^a^
	>60	2.07 (1.55–2.76)	<0.001	1.88 (1.29–2.74)	0.001^a^
Sex (M/F)		1.51 (1.28–1.77)	<0.001	1.43 (1.16–1.77)	0.001^a^
Comorbidities	Diabetes	0.78 (0.61–1.00)	0.054	0.72 (0.52–1.02)	0.065^b^
	CVD	0.89 (0.71–1.12)	0.33	0.86 (0.63–1.18)	0.37^b^
	Kidney disease	0.93 (0.62–1.41)	0.76	0.80 (0.46–1.38)	0.42^b^
	Liver disease	1.48 (0.94–2.32)	0.08	1.52 (0.85–2.73)	0.15^b^
	IDD	0.93 (0.57–1.50)	0.77	0.82 (0.43–1.55)	0.55^b^
	CPD	1.39 (1.002–1.93)	0.04	1.75 (1.14–2.68)	0.009^b^
Symptoms	Cough	0.76 (0.64–0.89)	0.001	0.75 (0.61–0.93)	0.011^b^
	Dyspnea	2.08 (1.76–2.46)	<0.001	2.00 (1.60–2.48)	<0.001^b^
	Myalgia	0.62 (0.52–0.77)	<0.001	0.81 (0.63–1.04)	0.11^b^
	Sore throat	0.79 (0.58–1.07)	0.13	0.91 (0.60.1.38)	0.63^b^
	Diarrhea	0.88 (0.61–1.27)	0.51	1.15 (0.72–1.84)	0.53^b^
	Headache	0.62 (0.46–0.84)	0.003	0.72 (0.48–1.08)	0.11^b^

Abbreviations: CPD, chronic pulmonary disease; CVD, cardiovascular disease; ICU, intensive care unit; IDD, immune deficiency disease.

^a^ Adjusted with ICU admission, ventilator aid, comorbidities.

^b^ Adjusted with age, sex, ICU admission, ventilator aid.

## 4 DISCUSSION

The coincidence of malignancy in COVID‐19 patients significantly increases the rate of mortality. However, among comorbidities and symptoms, only CPD and dyspnea were associated with increased mortality. Furthermore, concomitant comorbidities, including liver disease and IDD, were 8 and 4.71 times greater in patients with malignancy compared to the non‐malignant group.

Cancer is a major health issue with a 5‐year prevalence of 0.38% in Iran.^22^ According to our results, 1.68% and 1.11% of our hospitalized SARS and RT‐PCR‐positive patients had cancer. Given that our data is representative of the hospitalized Iranian population, we can assume that patients with malignancy were not well protected as the prevalence of malignancy among hospitalized SARS patients is higher than the prevalence of malignancy in the total population. It was even higher than a meta‐analysis of hospitalized COVID‐19 patients that estimated the prevalence of malignancy as about 0.92% (95% CI 0.56%–1.34%).^23^

It seems that there are differences in the geographical distribution of Covid‐19 mortality in patients with malignancy among 31 provinces (Figure 1). We investigated the socio‐demographic index (SDI) and health development index (HDI) to find out the underlying causes of this diversity; however, none of them were utterly justifying the pattern of the diversity among provinces. Therefore, further investigations are needed to better illustrate the factors associated with the differences in mortality rates between provinces.^24^, ^25^

In this study, the overall mortality rate (28.95%) of Covid‐19 was higher among patients with malignancy than patients without malignancy. This finding is generally supported by previous studies indicating a range of 13% and 40.5% mortality rate among cancer patients.^26^ In a cohort study, the mortality of cancer patients with Covid‐19 was 40.5% compared to a 28.5% mortality rate in those without cancer (hazard ratio [HR] 1.62, 95% CI 1.56–1.68; *p* < .001).^27^ Besides, the median time to severe events in cancer patients was significantly shorter in another study^28^ that needs to be further investigated in our country.

However, Rüthrich et al.^11^ reported no association between cancer and Covid‐19 mortality once other risk factors were adjusted. This variability among frequencies might be due to the heterogeneity in cancer types, different stages of cancers, treatment types, and whether the patients were on active treatments or not. To illustrate the differences of mortality between hospital‐acquired and nosocomial‐acquired Covid‐19 patients with cancer, Liang et al.^29^ contribute this to receiving active treatment among hospitalized cancer patients. On the contrary, other studies had firmly rejected any worsening influence of treatments on the outcome of these patients.^16^, ^30^ Another confounding factor might be the region in which the study was conducted, for example; a meta‐analysis revealed no significant risk of Covid‐19 infection in patients with colorectal cancer except for China.^31^ Additionally, the number of hospitalized patients during the pandemic may not be representative of the population before the pandemic in all regions.

Consistent with our study, age is previously reported as an independent risk factor for COVID‐19 prognosis.^32^, ^33^ This data was consistent with other studies reporting that older ages are associated with increased mortality rates in cancer patients.^16^, ^28^ In our study, patients aged more than 60 had 1.88 times higher odds of mortality compared to the 20–40 age group, which was consistent with other studies reporting that higher than 65‐year‐old were associated with higher mortality rates irrespective of other factors such as tumor stage.^34^ However, a study reported that age >65 was not an independent risk factor in hospitalized cancer patients' mortality.^29^ Our study also revealed that among young ages of <20 years Covid‐19 patients with malignancy, the mortality rate was not significantly different from the reference age. Given the likelihood of declined immune competence in older ages, which may deteriorate the immunosuppressed state in those with malignancy, they are more susceptible to Covid‐19 complications like acute respiratory distress syndrome and death.^35^

Consistent with previous studies, the male gender was associated with increased disease mortality in both groups of patients with and without malignancy.^36^, ^37^ However, in studies on hematologic malignancies and lung cancer, the mortality rate was not associated with the male gender.^38^, ^39^ It has also been reported that seropositivity against COVID‐19 was higher in female cancer patients, along with greater rates of seropositivity in gynecologic and breast cancers.^19^ Regarding the underlying mechanisms, studies have mentioned differences in lifestyle, behavior, and comorbid disorders between sexes. Additionally, experimental studies have indicated estrogen receptor signaling as a protective factor; also, sex steroids are thought to be involved in immune responses to COVID‐19.^40^, ^41^

Having the lungs as the main victim organ of COVID‐19 in mind, the underlying lung disorders makes them more vulnerable to potential damage of the disease.^42^ It is pivotal that only the presence of CPD in cancer patients was associated with an increased mortality rate among studied comorbidities. As studied in a meta‐analysis, the prevalence of COPD is estimated to be around 4% among patients with Covid‐19, which significantly increases the risk of mortality.^43^ In this line, a study mentioned that Covid‐19 patients with lung cancer had remarkably higher comorbid conditions, including COPD and asthma, compared to those without cancer.^39^ However, there is heterogeneity between studies on the impacts of comorbidities on COVID‐19 mortality. In the study of Garassino et al.,^8^ the presence of any comorbidities was correlated with increased mortality in univariate analysis; however, this association was not significant in multivariate analysis. Having that in mind, cancer patients have a greater chance of developing comorbidities that put them at a higher risk for Covid‐19 complications.^44^

In this study, the frequency of comorbid disorders among malignant patients from highest to lowest were CVDs, DM, CPD, kidney disease, liver disease, and IDD. Besides, except for DM and CVD, the frequency of other comorbidities was higher in hospitalized and Covid‐19 positive patients with malignancy.

According to the study by Lee et al.,^16^ concurrent comorbidities such as CVDs increased the chance of mortality. In contrast to our findings, a study conducted on 10 hospitals in Daegu Metropolitan City, Korea, revealed that concurrent malignancy with pre‐existing CVD or cardiovascular risk factors (CVRFs) resulted in greater mortality in Covid‐19 patients compared to those without CVD or CVDRFs.^45^ However, consistent with our results on diabetic patients with malignancy, a nationwide cohort study on solid cancer patients with Covid‐19 showed that the rate of diabetes was not significantly different among survivors and deceased patients.^7^

Interestingly, the prevalence of IDD and liver disease in COVID‐19 positive cases were 8 and 4.71 times greater in patients with malignancy compared to the non‐malignant group, although these comorbidities did not increase mortality rates among these patients. This might be interpreted as higher concomitance of these comorbidities, for example, chemotherapy‐induced liver injury or increased cancer incidence in patients with pre‐existing IDD.^37^, ^38^

On the contrary, a previous registry study on COVID‐19 cancer patients revealed that either chronic liver disease or cirrhosis was not significantly different between cancer and non‐cancer groups.^11^ However, only cirrhosis was included in our study due to the lack of detailed information on other liver disorders. Other meta‐analyses on the association of kidney disease with Covid‐19 revealed that kidney disease is associated with increased disease severity^43^, ^46^ and mortality.^43^ In line with our findings, the rate of concurrent chronic kidney disease with malignancy was not significantly different between survived and deceased patients in the study of Liang et al.^29^ However, the heterogeneity between studies might be due to the differences between the denominators of each of these studies.

Regarding the odds for mortality, dyspnea was associated with increased mortality in patients with malignancy. On the other hand, the cough was a protective factor for death in COVID‐19 cancer patients that might be associated with the presence of cough as a common symptom in mild and moderate COVID‐19 cases.^47^ Additionally, the relation of each symptom and disease severity is controversial. In the previously mentioned study by Liang et al.,^29^ none of the signs and symptoms were significantly different between survived and deceased cancer patients with Covid‐19. In a case‐control study on 185 COVID‐19 patients (92 patients with cancer vs. 92 healthy people) conducted in Iran, the frequency of fever and dry cough was reported to be significantly lower in cancer patients. However, the differences in diarrhea and headache were not significant between groups.^9^ The variations in the disease manifestation, data gathering, and reporting symptoms might be the reason for this heterogeneity.

As the most critical implication, our findings emphasize the emergent need for the timely mobilization of resources provide special services for at‐risk groups. According to this approach, cancer patients are one of the high‐risk groups that should be given special attention in the priority of allocating hospital services and preventive programs such as vaccines. In addition, regarding the fact that the humoral response in some groups of these patients after vaccination might be compromised, it is pivotal to follow the responses of these patients to manage the vaccination programs. For example, it has been reported that seroconversion rates following COVID‐19 vaccination in patients with cancer might be compromised, especially in older patients, those with hematological malignancies, and chemotherapy receivers, and these patients should take appropriate caution even after vaccination.^48^

This study was conducted on a nationwide data registry representative of the Iranian COVID‐19 hospitalized population, discussed the issue of cancer in these patients for the first time in Iran, and presented the mortality in different provinces. It had some limitations, such as the self‐reported history of cancer diseases, not having the status of receiving active anti‐cancer treatment, and the cancer type.

We tried to design a representative study with the lowest selection bias. In this study, all COVID‐19 hospitalized patients' data was gathered throughout the country. Given the immunocompromised state of patients with malignancy and their vulnerability to Covid‐19 complications, collecting data on the comorbidities, especially CPD, and determining mortality risk factors, can build on a better clinical view and help clinicians make decisions about the management and treatment of these cases accurately. For instance, according to our findings, concurrent CPD with COVID‐19 brings a higher priority for treatment and timely management. Furthermore, the findings of this study could be helpful for policymakers to develop a pandemic management plan and make us ready for potential future attacks of infectious diseases.

## AUTHOR CONTRIBUTIONS

*Conceptualization*, N.R., D.J., and O.T.; *Methodology*, S.K., N.R., M.K., and A.B.; *Investigation*, S.H.; *Software*, A.G.; *Formal Analysis*, M.K., S.K., and E.G.; *Writing—Original Draft*, M.K., A.B.; *Writing—Review & Editing*, S.H., S.M., R.H., A.D., A.K., and S.D.; *Visualization*, A.G., E.G., E.P., and R.W.; *Supervision*, N.R. and S.D.; *Project Administration*, S.D.

## CONFLICT OF INTEREST

The authors have stated explicitly that there are no conflicts of interest in connection with this article.

## ETHICS STATEMENT

This study was approved by the Ethical Committee of National Institute for Medical Research Development (ID; NIMAD.REC.1399.185).

## Data Availability

The data that support the findings of this study are available from the corresponding author, Shirin Djalalinia, upon reasonable request.
